# Efgartigimod beyond myasthenia gravis: the role of FcRn-targeting therapies in stiff-person syndrome

**DOI:** 10.1007/s00415-023-11970-1

**Published:** 2023-09-08

**Authors:** Vincenzo Di Stefano, Paolo Alonge, Nicasio Rini, Massimiliano Militello, Antonino Lupica, Angelo Torrente, Filippo Brighina

**Affiliations:** Department of Biomedicine, Neuroscience, and Advanced Diagnostic (BIND), University of Palermo, Via del Vespro, 143 90127 Palermo, Italy

**Keywords:** Efgartigimod, Stiff-person syndrome, Anti-GAD antibody, Glutamic acid decarboxylase, FcRn, Myasthenia Gravis, SPS-ADL

## Abstract

Stiff-person syndrome (SPS) is a rare autoimmune neurological disorder characterized by high titers of antibodies against glutamic acid decarboxylase (GAD) causing impaired GABAergic inhibitory neurotransmission. To date, there is not a defined therapy for such condition, but immunomodulating therapies, such as plasma exchange, intravenous immunoglobulins, and rituximab, have been widely used in clinical practice. However, the efficacy and tolerability of these treatments is not well established. Efgartigimod, a new neonatal Fc receptor (FcRn) blocker, is a human IgG1 antibody Fc fragment engineered with increased affinity for FcRn binding, leading to a reduction in IgGs levels, including pathogenic IgG autoantibody showing promising results in neurological autoimmune disorders and has been approved for the treatment of AChR-seropositive generalized myasthenia gravis (MG). In this study, we report and describe the first data on treatment with efgartigimod in three patients affected by both AChR-seropositive generalized MG and anti-GAD-seropositive SPS. Patients were followed since the start of efgartigimod and for the whole treatment period (12 weeks). MG symptoms were assessed with the “MG activity of daily living score” and the Quantitative Myasthenia Gravis score, while SPS ones were assessed with the “SPS activity of daily living score”; muscle strength was assessed with the Medical Research Council Sum score; the overall disability from MG and SPS was assessed by the modified Rankin Scale. All patients showed an improvement in symptoms of both SPS and MG after 2 cycles of treatment. Our data suggest that efgartigimod may be considered as a candidate drug for SPS and other autoantibody-mediated neurological disorders.

**Supplementary Information:**

The online version contains supplementary material available at 10.1007/s00415-023-11970-1.

## Introduction

Stiff-person syndrome (SPS) is an autoimmune condition caused by antibodies targeting several components of the inhibitory synapse in the spinal cord, with glutamic acid decarboxylase (GAD) antibodies being the predominant immune marker [1]. Epidemiological studies are scarce, but it is esteemed that SPS affects approximately 1 patient per million population per year and is usually more prevalent among women [1]. The syndrome results from reduced GABAergic transmission caused by GAD antibodies [1–3]. Indeed, GAD is an intracellular enzyme whose physiologic function is the decarboxylation of glutamate to gamma-aminobutyric acid (GABA), the main inhibitory neurotransmitter within the central nervous system [3]. The mainstay of the diagnosis relies on the detection of high titers of GAD Ab in serum and/or on their detection in patients’ cerebrospinal fluid [1]. Clinically, patients suffer from several neurological symptoms that are expression of an impaired GABAergic transmission in the central nervous system: pain, hyperexcitability, exaggerated startle response, ataxia, respiratory failure, with severe disability and frequent admission to intensive care units. Moreover, as it often happens for autoimmune diseases, SPS is frequently associated with other autoimmune conditions, such as thyroiditis, myasthenia gravis (MG), and psoriasis [4]. Despite the partially known pathogenetic mechanisms underlying SPS, unfortunately there is no defined therapy yet. High doses of intravenous Ig (IVIg), plasma exchange, and immunoadsorption are frequently used in the management of severe autoimmune diseases mediated by pathogenic IgG autoantibodies [5–7]. Such IgG modulating approaches can obtain a satisfactory clinical response in autoimmune diseases (including neurological ones), but are quite frequently associated with some severe adverse reactions and a substantial burden for patients. Hence, IVIg is liberally used as chronic therapy in SPS even if with limited efficacy data [1, 4, 7]. Furthermore, there are few cases of SPS treated with rituximab, but without clear results [1]. Indeed, due to the rarity of SPS, treatment schemes and predictors of response are poorly defined, highlighting the unmet need for multicentric prospective trials. As a result, SPS appears to date as a progressively disabling disease with no effective treatment [4].

Targeting the neonatal Fc receptor (FcRn) presents an innovative and potentially more effective, safer, and more convenient alternative for clearing pathogenic IgGs [8]. Indeed, FcRns recycle IgGs by preventing their lysosomal degradation. As this process also enhances the half-life of pathogenic auto-IgGs, several inhibitors of the IgG-FcRn interface have been conceived to treat autoimmune diseases [9]. Of interest, efgartigimod (ARGX-113), a new FcRn blocker, is a human IgG1 Fc fragment engineered to reduce pathogenic IgG autoantibody levels showing promising results in neurological autoimmune disorders, such as MG. Indeed, a phase 2 trial was carried out in 2019 with good results in MG patients [10], and then, a multicentre, randomized, placebo-controlled, phase 3 trial was conducted even in patients with generalized MG showing good efficacy and tolerability [11]. Finally, efgartigimod received FDA approval in December of 2021 and EMA approval in August 2022 for AChR-seropositive generalized MG [12, 13]. On this perspective, FcRn-targeting offers a relevant opportunity to treat SPS patients by reducing anti-GAD IgG Ab levels without significant immunosuppression or apheresis [4, 14]. In this study, we describe the first data of efgartigimod in patients affected by both AChR-seropositive generalized MG and anti-GAD-seropositive SPS.

## Methods

### Ethical statement

The study was conducted in accordance with the Declaration of Helsinki and approved by the Ethics Committee of “Policlinico Paolo Giaccone”, Palermo, Italy (Palermo I, protocol code 11/2022, approved on 12 December 2022). Informant consent was obtained for each participant.

### Patient’s population

The diagnosis of MG was made according to the following criteria: diffuse weakness, with or without ocular or respiratory involvement, together with either abnormal levels of anti-AChR-Ab or pathological neurophysiological findings (i.e., decremental U-shaped response at 3-Hz repetitive nerve stimulation and/or increased jitter at single-fibre electromyography—SFEMG). We excluded patients suffering from any other neurological or inflammatory condition but SPS [6]. Patients with anti-MuSK-seropositive and double-seronegative MG have been excluded as well. The diagnosis of SPS was made according to the Dalakas’s criteria: stiffness in the axial muscles, prominently in the abdominal and thoracolumbar paraspinal ones, leading to a fixed deformity (hyperlordosis); superimposed painful spasms precipitated by unexpected noises, emotional stress, or tactile stimuli; confirmation of the continuous motor unit activity in agonist and antagonist muscles by electromyography; absence of neurological or cognitive impairments that could explain the stiffness; positive serology for GAD65 (or amphiphysin) autoantibodies, assessed by immunocytochemistry, western blot or radioimmunoassay; response to diazepam [15].

All patients were screened for the presence of thymoma by means of computed tomography or magnetic resonance imaging scanning of the mediastinum. Therapies with immunomodulatory regimens (steroids, immunosuppressants, monoclonal antibodies) were stable in the last six months. Patients did not receive any IVIg cycle in the last 3 months before efgartigimod, while plasma exchange or thymectomy in the last 12 months.

### Procedures

Treatment with Efgartigimod occurred within the “Expanded Early Access Program for Efgartigimod IV treatment in patients with generalized myasthenia gravis (GENERATIVE Protocol)”. Patients were followed since the start of efgartigimod and for the whole treatment period (at least 12 weeks). Efgartigimod (10 mg/kg) was administered as four infusions per cycle (one infusion per week). After each cycle there was a period of at least 4 weeks of follow-up. All patients received an initial cycle, with a second cycle administered after 4 weeks. Each patient underwent clinical evaluation every week during the infusion period and a week after the last infusion per cycle (T0, I7, I14, I21, and I28 for the first cycle, and T1, II7, II14, II21, II28, for the second cycle). The severity of MG was assessed with the “MG activity of daily living (MG-ADL) score” (patient-reported, physician-recorded outcome measure) and the Quantitative Myasthenia Gravis (QMG) score; the severity of SPS syndrome was assessed with the “SPS activity of daily living (SPS-ADL) score” (patient-reported, physician-recorded outcome measure), adapted to identify and measure the impact of SPS symptoms in everyday life (see supplementary material); patients’ muscle strength was assessed using the Medical Research Council (MRC) sum score (from 0, absence of movement, to 5, normal strength) calculated for the upper (deltoid, triceps, biceps, wrist and finger extensors, wrist and finger flexors) and lower limbs (iliopsoas, tibialis anterior, gastrocnemius, toe extensors, toe flexors); the overall disability from MG and SPS was assessed by the modified Rankin scale (mRS). Testing for autoantibodies (i.e., anti-AChR, anti-GAD) was performed by the radioimmunoassay method using a radio-receptor assay kit. The results were reported as positive for AChR if > 0.50 nmol/l, and positive for anti-GAD if > 5.0 UI/ml [16]. All the MG patients underwent total IgG, thyroid hormones and thyroid stimulating hormone serum testing, antinucleus antibodies testing, complete blood count, creatinine, blood urea nitrogen, liver transaminases, gamma glutamyl transferase, and total and fractionated bilirubin. Assessments with MRC sum score were performed at the start and then weekly for 4 weeks after initiation of each cycle (T0, I7, I14, I21, I28, for the first cycle, and at T1, I7, II14, II21, II28, for the second cycle, etc.), while MG-ADL, QMG, SPS-ADL, and mRS were performed at the start of treatment (T0) and after the end of the second cycle (a week after the last infusion of the cycles, i.e., I28, II28, or III28), as well as anti-GAD, and anti-AChR dosage.

### Statistical analysis

Continuous variables were reported as mean and standard deviation (SD) or by median (maximum, minimum) within squared brackets according to their distribution. Categorical variables were presented as numbers and relative percentages.

## Results

### Patient’s population

We found three patients affected by both SPS and AChR-seropositive generalized MG in a cohort of 213 MG patients regularly followed at the Neuromuscular outpatient clinic of “Policlinico Paolo Giaccone” of Palermo, Italy. Table 1 describes clinical data from three patients included in this study.

**Table 1:** Clinical data of patients affected by MG and SPS treated with efgartigimod

	Patient 1	Patient 2	Patient 3
Age (y)	31	46	59
Sex	F	F	F
Age at MG onset (y)	29	40	53
Age at SPS onset (y)	30	45	57
MG subtype	AChR-seropositive generalized	AChR-seropositive generalized	AChR-seropositive generalized
Thymic pathology	No	Thymic hyperplasia	Thymic residual
Comorbidity	Anxiety, bronchial asthma, Hashimoto’s thyroiditis, vitamin B12 deficiency	Anxiety and depression, Hashimoto’s thyroiditis, vitamin B12 deficiency	Anxiety and depression, Graves’ disease
Failed treatments	Azathioprine	Plasma exchange	Rituximab
IVIg cycles in the previous year	1	2	1

*MG* myasthenia gravis, *AChR* acetylcholine receptor, *IVIg* immunoglobulin

### Patient 1

The first patient was a 31-year-old girl who presented to our attention in 2021 with a severe onset of MG with easy fatigue and weakness, difficulty walking and keeping arms raised, difficulty in swallowing and chewing, occasional ptosis, and evening diplopia. Her medical history mentioned only bronchial asthma, Hashimoto’s thyroiditis, and anxiety-depressive syndrome. Clinical, serological, and neurophysiological assessments allowed a diagnosis of AChR-seropositive MG with spinal-predominant symptoms (MGFA IVA, MG-ADL 20). She was treated with IVIg as a rescue therapy with some clinical improvement and immunomodulating therapy with prednisone 1 mg/kg with unsatisfactory control on her symptoms. Azathioprine was started, but then discontinued for laboratory signs of liver damage. One year after the onset of MG, the patient complained of episodic memory deficit, attention deficit, startle reaction, balance difficulties with falls, diffuse pain, and spasms at limbs. Then, a brain MRI was performed and showed no abnormal finding. After a diagnostic workup, reduced vitamin B12 levels (145 pmol/l; normal values > 230 pmol/l) had been demonstrated, so she was supplemented with intramuscular injection without any improvement on her symptoms in two months. Hence, after a more comprehensive evaluation, a diagnosis of SPS was achieved through seropositive Anti-GAD Abs finding, together with electromyographic evidence of continuous motor unit activity in agonist and antagonist muscles. Unfortunately, the SPS symptoms associated with the severe generalized MG confined the patient in a wheelchair and she had to abandon work and university activities.

### Patient 2

The second patient was a 46-year-old woman who was diagnosed with MG for severe fatigue with diffuse weakness, ptosis, and diplopia (MGFA IIA, MG-ADL 9). Her medical history mentioned only Hashimoto’s thyroiditis and anxiety-depressive syndrome. She was treated with IVIg as a rescue therapy with significant clinical improvement and immunomodulation therapy with prednisone 1 mg/kg and Azathioprine. After 4 years from the onset of MG, the patient complained cognitive impairment, balance difficulties with falls, and diffuse pain. Nevertheless, MRI scan of the brain was unremarkable. She was admitted to a Neurology ward where reduced vitamin B12 levels (201 pmol/l; normal values > 230 pmol/l) have been demonstrated and promptly supplemented with intramuscular injection. However, ataxia and cognitive impairment persisted while she still complained of frequent spasms and significant pain. Also, history of falls, balance difficulty, spasms, startle response, increased tone with brisk reflexes, speech difficulty, ataxia, dyspnoea, dysphagia, in the presence of other autoimmune conditions (autoimmune thyroiditis and MG) suggested a clinical diagnosis of SPS. Hence, a more comprehensive evaluation allowed a diagnosis of SPS due to anti-GAD Abs finding in the serum and CSF, confirmed even by neurophysiological assessment. However, no significant response was achieved with IVIg (2 g/kg distributed in 5 days).

### Patient 3

The third patient was a 59-year-old woman who was diagnosed with MG with significant diagnostic delay. She was admitted to Neurology ward several times for diplopia, ptosis, dyspnoea, dysphagia, and fatigue, but in the absence of AChR-Ab was initially diagnosed as affected by functional neurological disorder. Her medical history mentioned only Graves’ disease. Some clinicians hypothesized a seronegative MG, and she was treated with pyridostigmine and prednisone with a good response on ptosis and diplopia. Due to a finding of thymic residual, she underwent thymectomy in 2019 and was treated with IVIg with clinical benefit. After reduction of the dosage of prednisone, anti-AChR-Abs were detected in three different samples, as well as a neurophysiological confirmation of MG diagnosis was obtained with SFEMG (MGFA IIb). However, after a couple of years she complained of balance difficulties with frequent falls and cognitive impairment associated with spasms. Clinical and neurophysiological examination showed the presence of an exaggerated startle response, increased muscle tone with brisk reflexes, and ataxia. MRI of the brain was unremarkable. Hence, a diagnosis of SPS was confirmed with seropositive anti-GAD Abs in the serum. After a significant response achieved with two cycles of IVIg, she did not benefit from further IVIg cycles. Azathioprine was then started, but it was stopped afterwards for nausea and vomiting. Even Rituximab 1000 mg was started with unsatisfactory response after 6 months.

### Treatment schedule

**Fig. 1:**
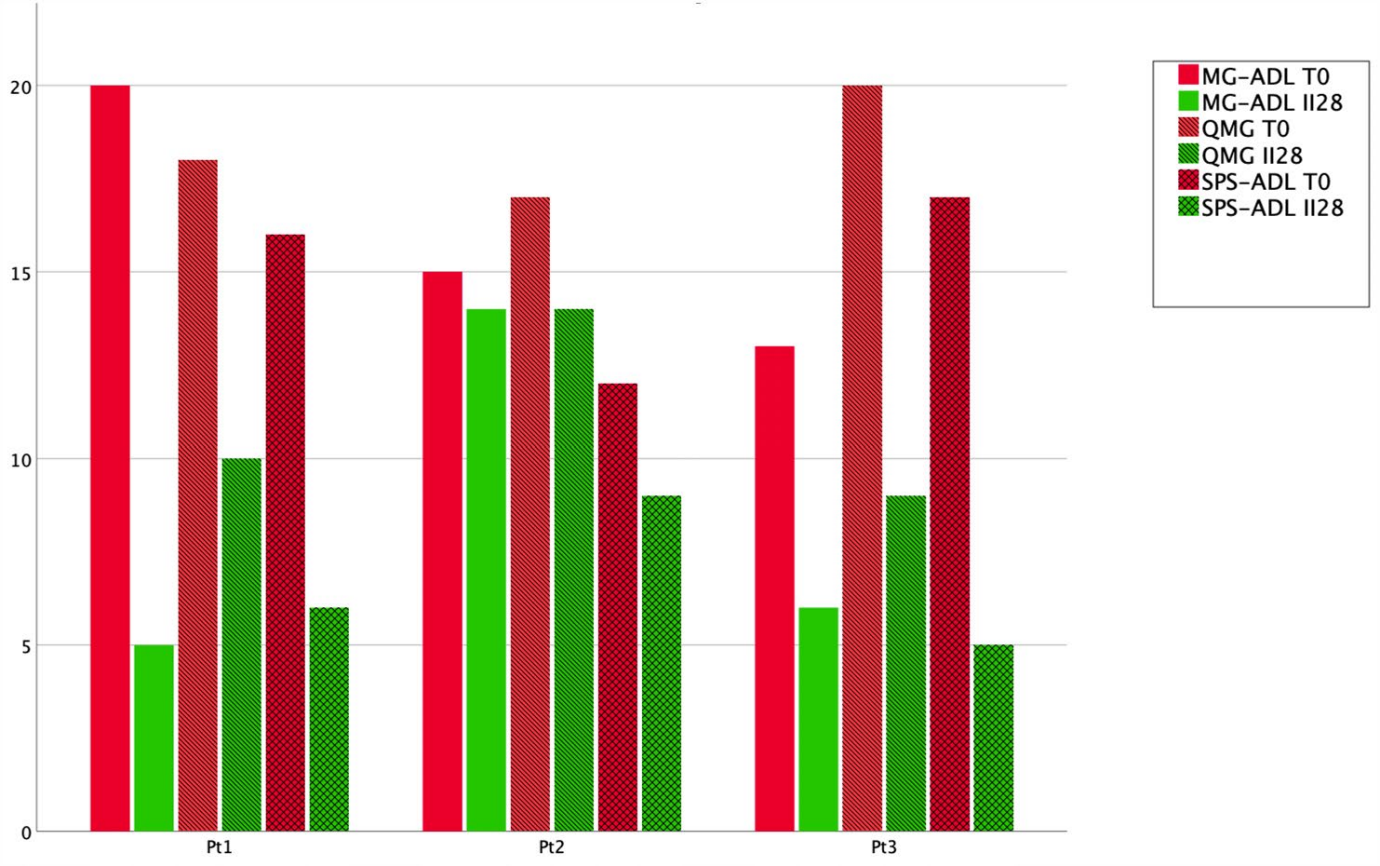
Comparison among single patients for MG-ADL, QMG, and SPS-ADL at the start of the first cycle (T0, red) and at the end second cycle of efgartigimod (II28, green). *MG-ADL* Myasthenia Gravis activity of daily living score, *QMG* Quantitative Myasthenia Gravis, *SPS-ADL* SPS activity of daily living

Efgartigimod was administered for two cycles in three patients affected by AChR-seropositive MG and anti-GAD-seropositive SPS. Overall, the treatment was well tolerated by patients, showing an effective response in both MG and SPS symptoms in all the subjects. Figure 1 describes the scores of the clinical scales at the start of the first cycle (T0) and at the end second cycle of efgartigimod (II28) for each patient. Patient 1 displayed a significant improvement in symptoms of both MG and SPS; patient 2 presented a stabilization of symptoms of MG and SPS with mild improvement. Patient 3 showed a very good response on both MG and SPS scales. Table 2 describes clinical and laboratory data before and after the treatment with efgartigimod.

**Table 2:** Comparison among clinical scales and laboratory data before and after two cycles of treatment with efgartigimod

	Baseline (mean ± standard deviation)	After second cycle of efgartigimod (mean ± standard deviation)
MG-ADL	16 ± 3.6	8.3 ± 6.6
MRC upper and lower limbs	103.3 ± 8.3	120.7 ± 11.5
QMG	18.3 ± 7.3	11.0 ± 8.4
SPS-ADL	15.0 ± 2.1	6.7 ± 2.1
Serum IgG (mg/dl)	1228.7 ± 508.7	727.0 ± 250.3
Anti-AChR-Ab (nmol/l)	0.86 ± 0.31	0.01 ± 0.001
Anti-GAD-Ab (UI/ml)	19.4 ± 7.7	1.15 ± 0.07
mRS	4 ± 1.0	3.3 ± 0.6

*MG-ADL* Myasthenia Gravis activity of daily living score, *MRC* Medical Research Council, *QMG* Quantitative Myasthenia Gravis, *SPS-ADL* SPS activity of daily living, *AChR* acetylcholine receptor, *GAD* glutamic acid decarboxylase, *mRS* modified Rankin scale

### Safety

Serum IgGs were recorded every week during the whole study period. As expected, a reduction of serum IgG was noticed but all patients maintained the recommended levels above 600 mg/dl for the whole study period (Table 2). Efgartigimod revealed to be safe without any serious adverse events in all patients. No infections or allergic reactions occurred during the infusions. Patient 1 reported a temporary mild lip burning after 24 h from the first infusion that was not reported after the following infusions. Patient 2 reported a transient asymmetric tremor in the left hand the day after the first administration that was not reported any more after the following infusions. All patients were satisfied of the rapid infusion protocol and the overall safety of efgartigimod.

### Myasthenia gravis assessment

**Fig. 2:**
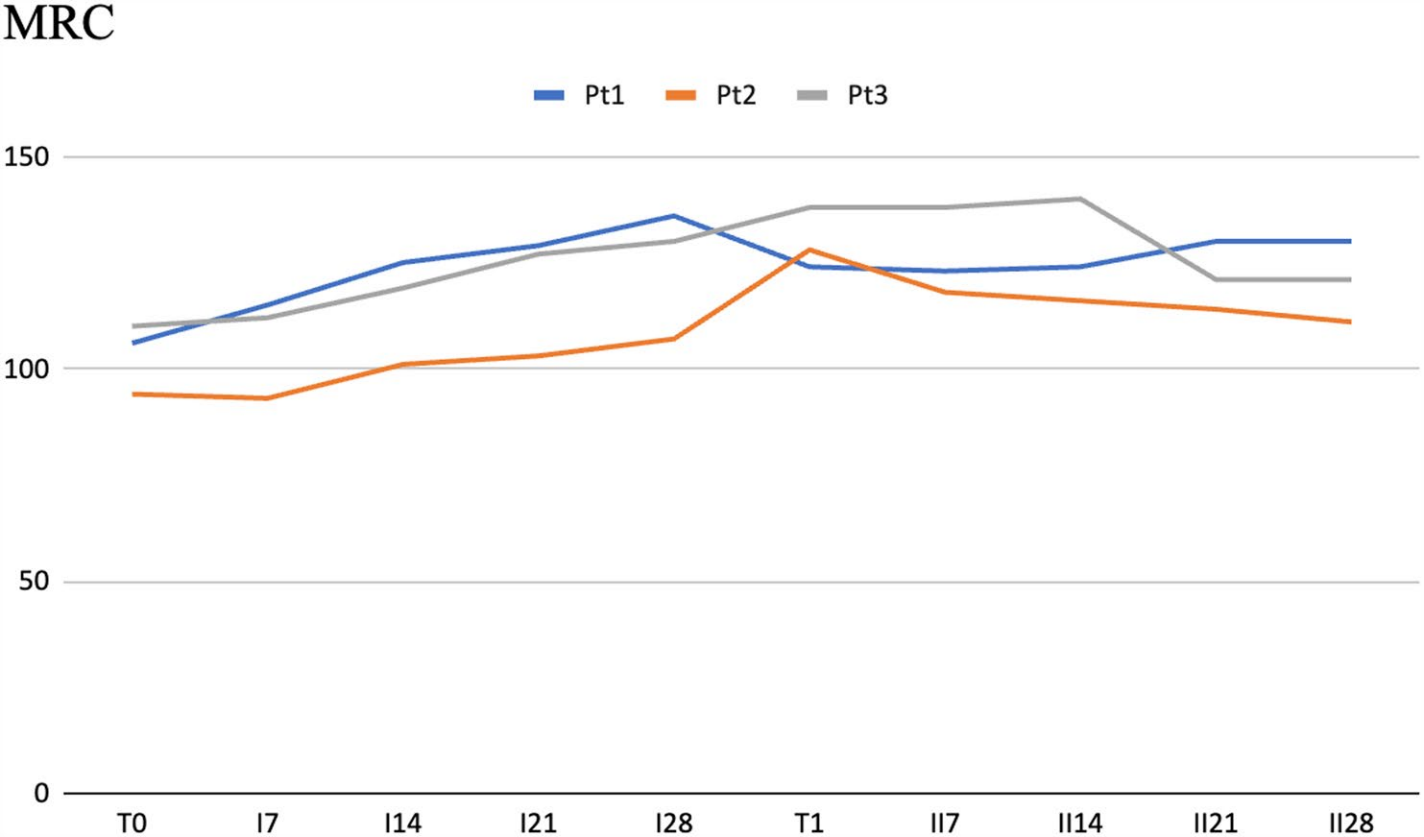
MRC sum score during the first and second cycle of efgartigimod. *MRC* Medical Research Council

Efgartigimod was effective in reducing the overall burden of MG. As expected, anti-AChR-Ab were reduced after treatment (Table 2). MG-ADL score was reduced of an average of seven points, with patients 1 and 3 losing more than 5 points from T0 to II28 (Fig. 1). Also, QMG score was reduced of six points (Table 2, Fig. 1). Finally, the improvement of MG symptoms was also demonstrated by increased strength assessed by MRC (Fig. 2).

### Stiff-person syndrome assessment

**Fig. 3:**
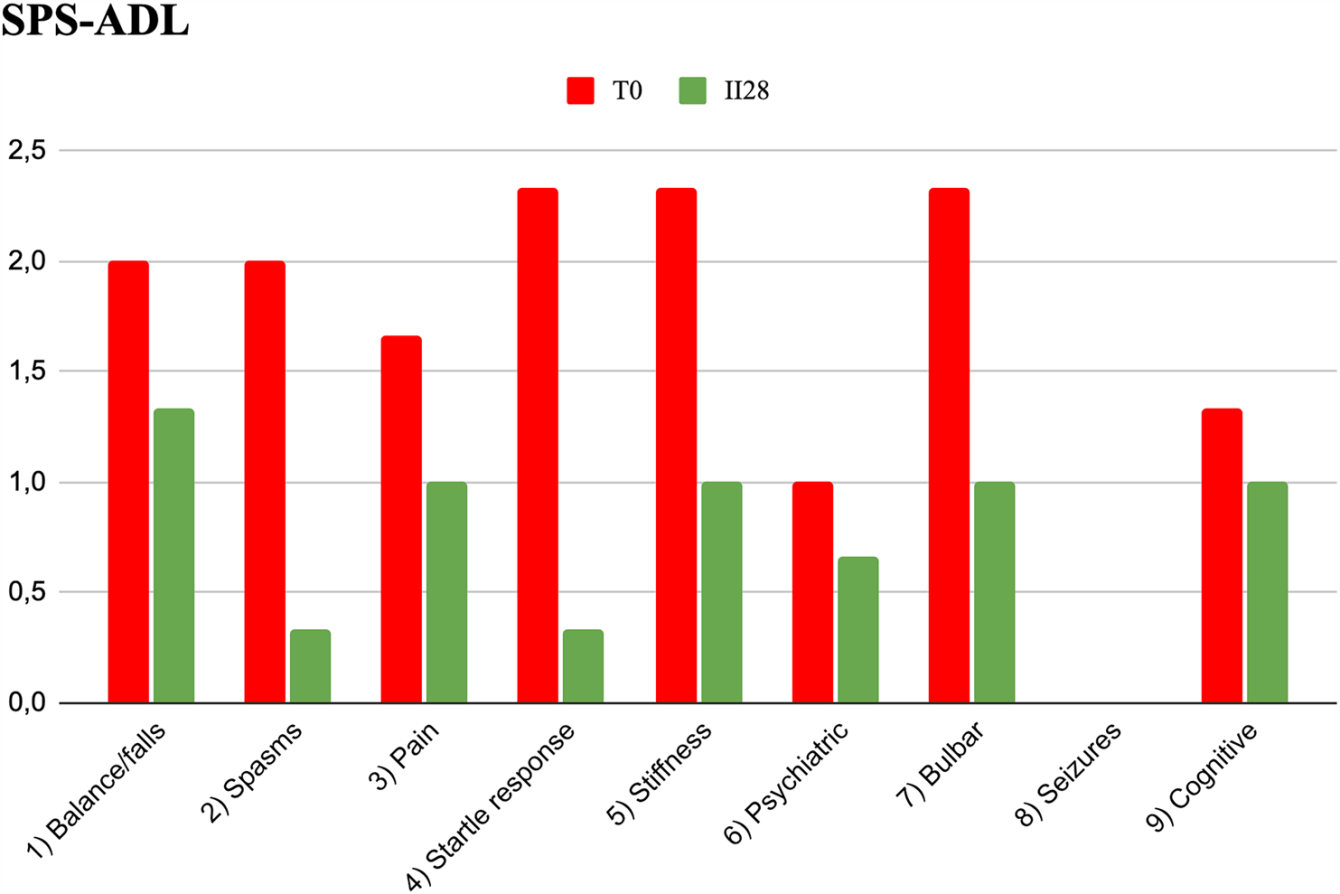
Mean SPS-ADL scores at the start of the first cycle (T0, red) and at the end second cycle of efgartigimod (II28, green). *SPS-ADL* SPS activity of daily living

Efgartigimod was effective in reducing the overall burden of SPS. As expected, *Anti-GAD*-Ab were reduced after treatment (Table 2). Supplementary material describes single score on SPS-ADL for each patient. Patient 1 experienced an overall reduction of nine points on SPS-ADL with a brilliant response. The benefit was evident on spasms and startle response that completely disappeared, while a reduction in falls and rigidity improved her quality of life and allowed ambulation. Also, after treatment the patient abandoned her wheelchair, and she felt more confident in herself and sometimes able to walk without aid for some distance. Patients 2 experienced mild improvement in leg stiffness with reduction in frequency of spasms and startle response; however, no effect was evident on pain and balance. Patient 3 showed the best improvement with a reduction of 12 points in SPS-ADL; spasms, exaggerated startle response, and pain disappeared, while a significant reduction was recorded for balance difficulties, stiffness, bulbar, and psychiatric symptoms. Considering the whole three patients, SPS-ADL score was reduced of an average 6 points. Figure 2 shows mean SPS-ADL score at T0 and II28. Items (2) spasms, (4) exaggerated startle response, (5) stiffness, and (7) bulbar symptoms were characterized by a more pronounced reduction after treatment with a mean reduction of more than two points (Fig. 3, Supplementary material).

### Immunosuppressive drugs and steroids

Table 3 compares treatments before and after treatment with efgartigimod. Immunosuppressive drugs dosage was not increased during treatment with efgartigimod and in no case any rescue therapy (plasmapheresis or immunoglobulins) was needed. Moreover, a reduction of the immunosuppressive drug was obtained for patients 1 and 2. Patient 1 reduced prednisone 20 mg after the first cycle and stopped it. Patient 2 stopped prednisone 15 mg and reduced azathioprine from 150 to 50 mg.

**Table 3:** Treatment of patients affected by MG and SPS treated with efgartigimod at the start of the first cycle (T0) and at the end second cycle of efgartigimod (II28)

	Patient 1	Patient 2	Patient 3
Ongoing treatments at the start of efgartigimod (T0)			
Pyridostigmine (mg)	240	240	240
Prednisone (mg)	20	15	0
Azathioprine (mg)	0	150	0
Ongoing treatments at the end of the second cycle of efgartigimod (II28)			
Pyridostigmine (mg)	240	240	240
Prednisone (mg)	0	0	0
Azathioprine (mg)	0	50	0

### Disability

An improvement of the overall disability was demonstrated in two patients (patients 1 and 2). Patient 1 presented severe disability at the beginning requiring constant nursing care and attention (mRS 5) and was able to attend her own bodily needs and walk without assistance at the end of the second cycle (mRS 3); patient 2 was able to walk unassisted at II28 (mRS from 4 to 3), while patient 3 presented stable moderate disability (mRS 3).

## Discussion

SPS is a rare autoimmune neurological condition characterized by central and peripheral hyperexcitability due to impaired GABAergic neurotransmission [1]. Patients affected by SPS experience progressive and severe neurological symptoms with increase in disability until they become bedridden or confined in a wheelchair. In most severe cases, patients can experience respiratory failure requiring admission to intensive care units [17, 18]. The management of SPS is based on immunosuppressants and antispastic drugs for chronic use, and IVIg and plasma exchange as rescue therapies [1, 19]. IVIg have been demonstrated as a safe and effective therapy to treat SPS exacerbations, but there are few data due to the rarity of the disease [2, 20]. Some researchers explored the role of rituximab in SPS with conflicting results [21, 22]; others employed subcutaneous immunoglobulins or autologous hematopoietic stem cell transplantation [23, 24]. Hence, the management of SPS is still empiric and based on single experiences, so a definite therapy is on demand.

Being an autoimmune disorder, SPS had been found to be associated with several autoimmune conditions including MG [25, 26]. In our neuromuscular outpatient clinic, we follow three patients affected by SPS and generalized MG. Hence, in this study, we aimed to verify the efficacy of efgartigimod in AChR-seropositive generalized MG patients with comorbid anti-GAD-seropositive SPS. The rationale for efgartigimod in SPS is the reduction of anti-GAD IgG Ab levels taking advantage of FcRn-targeting [4, 14]. Since its first evaluation in animal studies, efgartigimod improved muscle mass in mouse models for MuSK myasthenia gravis (MG) [27]. The phase 3 ADAPT study (NCT03669588) has recently demonstrated that efgartigimod, a novel FcRn inhibitor, is well tolerated in AChR-seropositive MG with significant improvements in MG-ADL and QMG scores [28]. Antagonizing FcRn using efgartigimod is safe and results in a specific, profound, and sustained reduction of serum IgG levels [8]. Hence, FcRn blockade is an emerging therapy in disease with indication to plasma exchange [29].

Data from the present study are in line with the registration study as all patients experienced an overall improvement on MG scales (Fig. 1) since the first two cycles of treatment. Moreover, efgartigimod revealed to be safe since patients reported no serious adverse event. As expected, AChR-Ab levels decreased, while a progressive increase in muscle strength was observed by MRC score increase (Fig. 2). Regarding SPS, anti-GAD antibodies decreased after the second cycle of efgartigimod. Of note, a significant effect was obtained as reported by the complete disappearance of spasms and startle response in patients 1 and 3. A mild reduction was reported also in falls and pain, but the benefit was less clear (Fig. 3). Also, a reduction of respiratory impairment and dyspnoea and dysphagia was also observed (Fig. 3), but it is difficult to say if they were consequent to a response to MG or SPS; studies focused on SPS patients without MG will clarify this issue. A mild improvement on pain, psychiatric symptoms, and cognitive impairment was also reported on SPS-ADL (Fig. 3), but there may be a role of secondary depression and anxiety in both perception of pain and cognitive function.

Furthermore, it should underline the role of efgartigimod as sparing drug: indeed, in two patients it was possible to stop prednisone after two cycles of efgartigimod. On this perspective, efgartigimod may be an alternative particularly for patients on long-term immunosuppressive regimens, especially patients on plasma exchange [14]. Indeed, efgartigimod might be administered at the first signs and symptoms of exacerbation or at a fixed schedule. The first approach aspires to tailored treatment minimizing exacerbations in patients with low disease burden; conversely, a fixed schedule (i.e., 1 cycle every 6 or 8 weeks) might aim to reduce the dosage of concomitant immunosuppressive therapies and the overall burden of the disease. However, controlled studies are needed to identify the best therapeutic scheme. We hypothesize that efgartigimod might be a candidate drug for SPS and other autoantibody-mediated neurological disorders, potentially increasing treatment options for these difficult neurological conditions.

### Limitations

This study presents several limitations. First, the small sample size is a main limitation. Indeed, this preliminary data on efgartigimod in SPS should be confirmed on large cohorts of patients in randomized and controlled studies. Moreover, a placebo effect cannot be excluded in both MG and SPS symptoms, especially due to some clinical overlap between the two conditions. Also, we cannot define the appropriate treatment schedule for efgartigimod in SPS, as patients have been treated after at exacerbations as for the ADAPT study, but not at a fixed schedule. Finally, future controlled studies on large cohorts of patients affected by isolated SPS are needed to better define the opportune frequency of treatment regimens and long-term safety and efficacy.

### Supplementary Information

Below is the link to the electronic supplementary material.

Supplementary file1 (DOCX 22 KB)

## Data Availability

Data are available from the corresponding author upon reasonable request.
