# Supplementary material for: Efgartigimod beyond myasthenia gravis: the role of FcRn-targeting therapies in stiff-person syndrome
Source: J Neurol. 2023 Sep 8;271(1):254–62. doi: 10.1007/s00415-023-11970-1 (PMC10769952; doi:10.1007/s00415-023-11970-1)
Supplement: Supplementary file 1 — Supplementary file1 (DOCX 22 KB) [file 415_2023_11970_MOESM1_ESM.docx]

**Stiff-person syndrome activity of daily living (SPS-ADL)**

Your generalized SPS symptoms can vary from day to day. Painting a clear picture of how they affect you over time is the best way that you and your doctor can ensure that you receive the best care possible. This assessment tool allows you to measure the symptoms that most affect your daily living. Fill out this form with your doctor.

Form should take just 10 minutes to complete. Simply give yourself a score (from 0-3) for each activity listed and add the results

1) Did you experience **balance difficulty** **or falls** in the last 7 days?

0 No balance difficulties

1 mild balance difficulties (mild instability with less than 2 falls)

2 moderate balance difficulties (significant instability with more than 2 falls or need to use precautions or aids)

3 severe balance difficulties (inability to maintain upright position with or without falls or patient in a wheelchair/bedridden)

2) Did you experience **spasms** in the last 7 days?

0 No spasms

1 mild (rare spasms without limitation in daily activity)

2 moderate (frequent spasms with some limitation in daily activity)

3 severe (very frequent spasms with significant limitation in daily activity)

3) Did you experience **pain** in the last 7 days?

0 No pain

1 mild (pain of low intensity without limitation in daily activity)

2 moderate (pain of moderate intensity with some limitation in daily activity)

3 severe (constant pain with significant limitation in daily activity)

4) Did you experience **exaggerated startle response** in the last 7 days?

0 No startle response.

1 mild (rare episodes of exaggerated startle response for high intensity stimuli)

2 moderate (frequent, but not daily, episodes of exaggerated startle response for moderate intensity stimuli)

3 severe (daily episodes of exaggerated startle response for mild or moderate intensity stimuli)

5) Did you experience **stiffness or increased muscular tone** in the last 7 days?

0 No stiffness.

1 mild (limited stiffness to one limb or mild rigidity and increased muscular tone without interference with movements)

2 moderate (diffuse stiffness to more than one limb and/or trunk, or moderate rigidity and increased muscular tone with some interference with movements)

3 severe (diffuse stiffness to more than one limb and/or trunk, or moderate rigidity and increased muscular tone with relevant interference with movements)

6) Did you experience **psychiatric symptoms** (i.e., mood changes, psychosis, hallucinations) in the last 7 days?

0 No psychiatric symptoms.

1 mild (occasional mood changes with anxiety, depression or agitation without interference with daily activities and social functions)

2 moderate (frequent mood changes with anxiety, depression or agitation with some interference with daily activities and social functions)

3 severe (daily mood changes or agitation or hallucinations with anxiety, depression with significant interference with daily activities and social functions)

7) Did you experience **bulbar symptoms** (i.e., dysphagia, dyspnea, dysphonia) in the last 7 days?

0 No bulbar symptoms.

1 mild (occasional dysphagia, or dyspnea, or dysphonia without interference with daily activities and social functions)

2 moderate (frequent dysphagia, or dyspnea, or dysphonia with some interference with daily activities and social functions)

3 severe (constant dysphagia, or dyspnea, or dysphonia with some interference with daily activities and social functions)

8) Did you experience **seizures** (i.e., convulsions, epilepsy) in the last 7 days?

0 No seizures.

1 mild (single crisis without witnessed loss of consciousness)

2 moderate (more than one crisis with witnessed loss of consciousness)

3 severe (more than 2 crises with witnessed loss of consciousness)

9) Did you experience **cognitive disfunction** (i.e., confusion, memory loss, attention deficits, difficulty in concentration) in the last 7 days?

0 No cognitive disfunction.

1 mild (single episodes of memory loss or some difficulty in concentration)

2 moderate (frequent episodes of memory loss or moderate difficulty in concentration)

3 severe (very frequent episodes of memory loss or impossibility to concentrate)

**Patient 1: SPS-ADL at time 0 (before treatment with efgartigimod) and II28 (a week after the second cycle of efgartigimod)**

| ***Item*** | ***T0*** | ***II28*** |
| --- | --- | --- |
| 1) Did you experience **balance difficulty** **or falls** in the last 7 days? | 2 | 1 |
| 2) Did you experience **spasms** in the last 7 days? | 2 | 0 |
| 3) Did you experience **pain** in the last 7 days? | 2 | 1 |
| 4) Did you experience **exaggerated startle response** in the last 7 days? | 3 | 0 |
| 5) Did you experience **stiffness or increased muscular tone** in the last 7 days? | 3 | 2 |
| 6) Did you experience **psychiatric symptoms** (i.e., mood changes, psychosis, hallucinations) in the last 7 days? | 0 | 0 |
| 7) Did you experience **bulbar symptoms** (i.e., dysphagia, dyspnea, dysphonia) in the last 7 days? | 2 | 1 |
| 8) Did you experience **seizures** (i.e., convulsions, epilepsy) in the last 7 days? | 0 | 0 |
| 9) Did you experience **cognitive disfunction** (i.e., confusion, memory loss, attention deficits, difficulty in concentration) in the last 7 days? | 2 | 1 |
| Total score | 16 | 6 |

**Patient 2: SPS-ADL at time 0 (before treatment with efgartigimod) and II28 (a week after the second cycle of efgartigimod)**

| ***Item*** | ***T0*** | ***II28*** |
| --- | --- | --- |
| 1) Did you experience **balance difficulty** **or falls** in the last 7 days? | 2 | 2 |
| 2) Did you experience **spasms** in the last 7 days? | 2 | 1 |
| 3) Did you experience **pain** in the last 7 days? | 1 | 2 |
| 4) Did you experience **exaggerated startle response** in the last 7 days? | 2 | 1 |
| 5) Did you experience **stiffness or increased muscular tone** in the last 7 days? | 1 | 0 |
| 6) Did you experience **psychiatric symptoms** (i.e., mood changes, psychosis, hallucinations) in the last 7 days? | 1 | 1 |
| 7) Did you experience **bulbar symptoms** (i.e., dysphagia, dyspnea, dysphonia) in the last 7 days? | 2 | 1 |
| 8) Did you experience **seizures** (i.e., convulsions, epilepsy) in the last 7 days? | 0 | 0 |
| 9) Did you experience **cognitive disfunction** (i.e., confusion, memory loss, attention deficits, difficulty in concentration) in the last 7 days? | 1 | 1 |
| Total score | 12 | 9 |

**Patient 3: SPS-ADL at time 0 (before treatment with efgartigimod) and II28 (a week after the second cycle of efgartigimod)**

| ***Item*** | ***T0*** | ***II28*** |
| --- | --- | --- |
| 1) Did you experience **balance difficulty** **or falls** in the last 7 days? | 2 | 1 |
| 2) Did you experience **spasms** in the last 7 days? | 2 | 0 |
| 3) Did you experience **pain** in the last 7 days? | 2 | 0 |
| 4) Did you experience **exaggerated startle response** in the last 7 days? | 2 | 0 |
| 5) Did you experience **stiffness or increased muscular tone** in the last 7 days? | 3 | 1 |
| 6) Did you experience **psychiatric symptoms** (i.e., mood changes, psychosis, hallucinations) in the last 7 days? | 2 | 1 |
| 7) Did you experience **bulbar symptoms** (i.e., dysphagia, dyspnea, dysphonia) in the last 7 days? | 3 | 1 |
| 8) Did you experience **seizures** (i.e., convulsions, epilepsy) in the last 7 days? | 0 | 0 |
| 9) Did you experience **cognitive disfunction** (i.e., confusion, memory loss, attention deficits, difficulty in concentration) in the last 7 days? | 1 | 1 |
| Total score | 17 | 5 |
